# Automated Microfluidics‐Assisted Hydrogel‐Based Wet‐Spinning for the Biofabrication of Biomimetic Engineered Myotendinous Junction

**DOI:** 10.1002/adhm.202402075

**Published:** 2024-09-23

**Authors:** Marina Volpi, Alessia Paradiso, Ewa Walejewska, Cesare Gargioli, Marco Costantini, Wojciech Swieszkowski

**Affiliations:** ^1^ Faculty of Materials Science and Engineering Warsaw University of Technology Warsaw 02‐507 Poland; ^2^ Department of Biology University of Rome Tor Vergata Rome 00133 Italy; ^3^ Institute of Physical Chemistry Polish Academy of Sciences Warsaw 01‐224 Poland

**Keywords:** 3D bioprinting, biofabrication, hydrogel fibers, microfluidics‐assisted wet‐spinning, myotendinous junction, skeletal muscle tissue engineering

## Abstract

The muscle‐tendon junction (MTJ) plays a pivotal role in efficiently converting the muscular contraction into a controlled skeletal movement through the tendon. Given its complex biomechanical intricacy, the biofabrication of such tissue interface represents a significant challenge in the field of musculoskeletal tissue engineering. Herein, a novel method to produce MTJ‐like hydrogel yarns using a microfluidics‐assisted 3D rotary wet‐spinning strategy is developed. Optimization of flow rates, rotational speed, and delivery time of bioinks enables the production of highly compartmentalized scaffolds that recapitulate the muscle, tendon, and the transient MTJ‐like region. Additionally, such biofabrication parameters are validated in terms of cellular response by promoting an optimal uniaxial alignment for both muscle and tendon precursor cells. By sequentially wet‐spinning C2C12 myoblasts and NIH 3T3 fibroblasts, a gradient‐patterned cellular arrangement mirroring the intrinsic biological heterogeneity of the MTJ is successfully obtained. The immunofluorescence assessment further reveals the localized expression of tissue‐specific markers, including myosin heavy chain and collagen type I/III, which demonstrate muscle and tenogenic tissue maturation, respectively. Remarkably, the muscle‐tendon transition zone exhibits finger‐like projection of the multinucleated myotubes in the tenogenic compartment, epitomizing the MTJ signature architecture.

## Graphical abstract

In this study, highly biomimetic myotendinous junction‐like hydrogel yarns are obtained by combining a microfluidics‐assisted 3D rotary wet‐spinning strategy with programmable syringe pumps. The multicellular scaffolds exhibit localized expression of skeletal muscle and tendon‐specific markers. Besides, the interface zone shows interpenetration of mature myotubes within the tenogenic extracellular matrix.

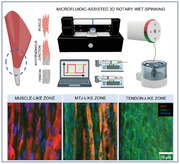

## 1 Introduction

The main function of the musculoskeletal system to generate movement relies on the muscle‐tendon unit (MTU), a biomechanical heterogenous interface structurally optimized to efficiently transfer the contractile force generated by muscles through tendons onto bone.^[^ ^1^ ^]^ The native MTU can be subdivided into three distinct regions: i) skeletal muscle, hallmarked by uniaxially oriented and multinucleated myofibers; ii) tendon, a highly organized connective tissue primarily composed of specialized fibroblasts (i.e., tenocytes and tenoblasts) surrounded by longitudinally aligned collagen fibers; and iii) a muscle‐tendon junction (MTJ), a specialized complex interface region where muscle myofibers form finger‐like projections, interdigitating with the tendon extracellular matrix (ECM).^[^ ^2^, ^3^, ^4^ ^]^ At the microscale, the effective connection between these tissues relies on a spectrum of intramembrane focal adhesion protein complexes, i.e., talin, vinculin, paxillin, and integrin, which connect the subsarcolemma to the extracellular laminin, anchoring the muscle cytoskeleton to the dense tendon ECM.^[^ ^5^ ^]^ Such an intertwined network is crucial as it provides an amplified contact area and a gradient of tissue composition, enabling a smooth transition in mechanical impedance and minimizing local stress concentrations.^[^ ^6^ ^]^

This sophisticated architecture, combined with its unique biomechanical demands, renders this tissue interface particularly susceptible to injuries.^[^ ^7^ ^]^ In fact, damages at the MTJ compromise the mechanical load distribution, leading to limited mobility and quality of life detriment.^[^ ^8^ ^]^ Besides traumatic injuries, potential harm to the muscle‐tendon interface predominantly arises from repeated overloading, commonly resulting from high‐impact athletic training or habitual overuse in the aging and senior demographic. Due to the extreme loads that the MTJ bears during movement, the most impacted regions are the rotator cuff, Achilles, and hamstring tendon.^[^ ^9^ ^]^ Severe alterations at the mechanical level can also result from damages occurring at the muscle or tendon proximate to the MTJ without a direct rupture. For instance, minor and localized tendon tears can also compromise the tissue interface by amplifying stress concentrations upon muscle contraction, catalyzing potential full‐thickness tears.

Although minor injuries might benefit from rest, severe tears or complete ruptures require surgical approaches.^[^ ^10^ ^]^ Conventional suture repairs are considered the gold standard treatment and generally allow for effective reattachment, albeit with significant fibrotic scar tissue formation, jeopardizing the biomechanical MTJ features and heightening reinjury risks.^[^ ^11^ ^]^ Moreover, untreated severe injuries can lead to muscle atrophy, complicating the physical reattachment process.^[^ ^12^ ^]^ Alternatively surgical strategies, such as autografts and allografts, are also well‐established. However, they are hindered by several limitations, including donor site morbidity, high clinical cost, biocompatibility, and poor integration to the host tissues.^[^ ^9^, ^13^ ^]^ Taken together, MTJ treatment and regeneration represent a significant therapeutic challenge.^[^ ^10^, ^14^ ^]^ Thus, innovative and functional therapeutic solutions are imperatively required, aiming to address both immediate repair and ensure long‐term functional recovery without predisposing the site to future damage. In this scenario, tissue engineering strategies provide a more promising alternative to the current clinical standard treatment. Innovative biofabrication methods should emulate the inherent biomechanical continuum while ensuring cellular alignment and guiding differentiation towards target tissue lineages.^[^ ^9^ ^]^ Recently, microfluidics wet‐spinning technologies gained attention as a biofabrication strategy to produce meter‐long hydrogel microfibers in a relatively short time, enabling the encapsulation of a wide range of different cell types and providing geometrical cues to direct cell anisotropic orientation within the constructs.^[^ ^15^, ^16^, ^17^ ^]^ Remarkably, individual fibers can also be assembled in more complex 3D structures using different methods, e.g., reeling or weaving.^[^ ^18^, ^19^ ^]^ Wet‐spinning techniques have been used as a successful tissue engineering platform for the fabrication of fibrous biological constructs, such as engineered skeletal muscle and tendon.^[^ ^20^, ^21^, ^22^, ^23^, ^24^ ^]^

Furthermore, the integration of microfluidic systems with 3D printing technologies showed significant potential in biofabricating the next generation of heterogeneous scaffolds for tissue engineering applications. In particular, microfluidic chips facilitate the simultaneous extrusion or on‐the‐fly switching among different bioinks by using programmable pumps or integrated valves. This approach enables the fabrication of multicellular and multimaterial constructs, thereby providing a more accurate representation of the intricate features of biological tissues.^[^ ^25^ ^]^ In this work, we developed a novel automated microfluidics‐assisted wet‐spinning method for the fabrication of a bicompartmentalized scaffold, which holds the biomimetic potential to reproduce in vitro the MTJ architecture. Such biofabrication strategy relies on a microfluidic head able to sequentially extrude multi‐cellular hydrogel core–shell microfibers, which are subsequently deposited on a rotating collecting drum. The resulting scaffold consists of highly aligned yarns with a smooth and cohesive transition among the muscle, MTJ, and tendon‐like region. The effect of various wet‐spinning parameters has been investigated to identify the optimal spinnability window and the degree of compartmentalization. Furthermore, the orientation of muscle and tendon precursor cells encapsulated within the microfibers has been investigated in terms of nuclei and F‐actin orientation to validate the potential to induce proper alignment as a first step requirement for proper muscle and tendon tissue maturation. Finally, cellular organization and expression of tissue‐specific markers have been analyzed to assess the scaffold's potential to serve as a platform to functionally replicate the intricate biological heterogeneity and architecture of the muscle‐tendon interface.

## 2 Results and Discussion

### 2.1 Microfluidics‐Assisted Wet‐Spinning of MTJ‐Like Hydrogel Yarns

**Figure 1:**
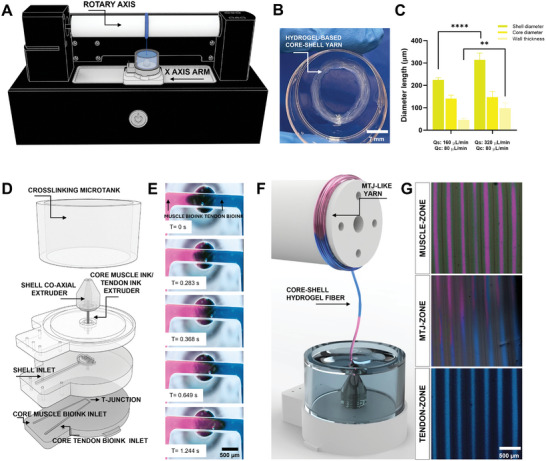
Schematics of 3D rotary wet‐spinning set up for the biofabrication of myotendinous junction (MTJ)‐like scaffolds. A) Rotatory microfluidic‐assisted 3D printer for the wet‐spinning of hydrogel core–shell bundles. B) Macroscopic image of a core–shell hydrogel bundle. Shell composition: 3% (w/v) alginate, core composition: 1.4% (w/v) fibrinogen + 0.2% (w/v) alginate. C) Fiber diameter, core diameter, and shell thickness for the selected shell flow rate (*Q*_s_) and core flow rate (*Q*_c_). D) Design of the microfluidic head used to fabricate MTJ‐like yarns. The core ink channels are equipped with a T‐junction to guarantee the sequential delivery of muscle‐like ink (m‐ink) and tendon‐like ink (t‐ink). E) Time‐lapse optical images of the T‐junction during the sequential delivery of m‐ink and t‐ink. F) Schematic of the microfluidic‐assisted wet‐spinning process of MTJ‐like yarns. G) Fluorescence images of core–shell fibers with graded core composition to generate three different scaffold regions: muscle, MTJ, and tendon. *n* = 5; significant differences: *p < 0.05, **p < 0.01, ***p < 0.001, and ****p < 0.0001.

In our recent work, we have reported a cutting‐edge biofabrication platform for the microfluidics wet‐spinning of engineered myo‐substitutes (**Figure** **1**A).^[^ ^26^ ^]^ Such platform consists of a 3D rotary wet‐spinning printer which is composed of three main components: i) a microfluidic printing head designed for core–shell hydrogel microfiber extrusion, ii) a rotating drum for hydrogel microfiber systematic collection and iii) an x‐axis motion arm for the consequentially automated assembly of 3D structures. In particular, the microfluidic printing head used in this study is characterized by three main inlets, two of which are designated to supply the core inks and the other for the fiber shell. These inlets are fluidically connected to the two compartments of a co‐axial extrusion nozzle, enabling the convergence of the core and shell inks into a defined core–shell flow configuration. Such composite bioinks are then extruded within a crosslinking bath microtank where an instantaneous ionic crosslinking occurs between the alginate constituents in the shell matrix and the calcium ions present in the bath, thus immobilizing the core/shell arrangement. Such design streamlines the sustained fabrication of core–shell hydrogel fibers, obviating the necessity for an additional crosslinker flow typically provided through multi‐co‐axial nozzles and ultimately reducing the complexity of the fiber formation process.^[^ ^27^ ^]^ Thanks to the mechanical support of the alginate shell, the proposed system enables the confinement of a broad spectrum of low‐viscosity bioink cores, which might otherwise be precluded from the 3D bioprinting process (Figure S1, Supporting Information). As a result of the rotational motion of the drum, the collected fibers were deposited to form an aligned structural layout, resulting in hydrogel core–shell microfiber‐based bundles characterized by pronounced anisotropic configurations (Figure 1B). This intrinsic property satisfied the architectural prerequisites for both skeletal muscle and tendon tissue engineering, mirroring the physiological orientation observed in native muscle and tendon biological matrices. In order to be compatible with the wet‐spinning process, the inks selection should simultaneously guarantee a rapid extrusion process and support cell growth, proliferation, and maturation into the target tissue. Accordingly, we selected for the shell‐ink a 3% w/w alginate and for the core‐ink a solution containing 1.4% w/w of fibrinogen and 0.2% w/w alginate, as previously reported.^[^ ^26^ ^]^ Another advantageous aspect of the proposed wet‐spinning bioprinter was the possibility to control, in certain ranges, the core and shell dimensions by simply tuning the flow rates of the respective inks. For example, as shown in Figure 1C, the increase of shell flow rate (*Q*s) values, while keeping fixed the core flow rate (*Q*_c_), resulted in an increased overall microfiber dimension and a thicker shell. Such technological features could be used to tune the dimensional configuration of the core–shell fibers in relation to the spinning biofabrication process and cell biocompatibility in terms of oxygen and nutrients diffusions. To recapitulate the architectural biocomplexity of the muscle‐tendon interface, the two core inlets were designed to converge into a T‐junction for the sequential and alternate delivery of the muscle‐like ink (m‐ink) and tendon‐like ink (t‐ink) (Figure 1D,E). By finely tuning Q_s_, Q_c,_ and rotational speed (RS), a tri‐compartmentalized heterogeneous hydrogel yarn could be generated; thus, encompassing distinct muscle, tendon, and MTJ regions (Figure 1F,G). Notably, the interface between the muscle and the tendon region presented a transitional pattern. This was attributed to the rapid alternation between the m‐ink and t‐ink, engendering a gradient architecture.

### 2.2 Optimization of Wet‐Spinning Parameters

**Figure 2:**
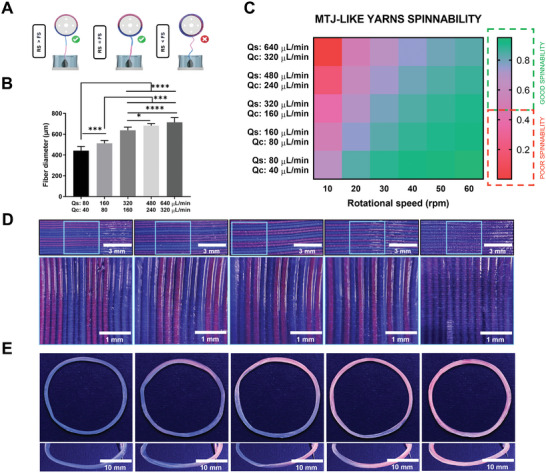
Schematics of myotendinous junction (MTJ)‐like scaffolds spinnability. A) MTJ‐like core–shell hydrogel yarns are successfully fabricated when the rotational speed (RS) of the collecting drum is higher or equal to the extruded fiber speed (FS). B) Diameter of core–shell microfibers wet‐spun at selected flow rates. C) Heat map of MTJ‐spinnability showing the combination of shell flow rate (*Q*_s_), core flow rate (*Q*_c_) and RS suitable for the fabrication of MTJ‐like scaffolds. D) One‐layered scaffold with different ratios of muscle‐like ink (m‐ink) and tendon‐like ink (t‐ink) core–shell microfibers obtained by tuning the m‐ink and t‐ink's delivery time (DT) within the MTJ‐like scaffolds spinnability window. E) MTJ‐like scaffolds with different percentages of m‐ink and t‐inks obtained by tuning the m‐ink and t‐ink's DT within the MTJ‐like scaffolds spinnability window. The core–shell fibers and the MTJ‐like core–shell yarns were obtained by using *Q*_s_ = 320 µL min^−1^ and *Q*_c_ = 160 µL min^−1^. *n* = 5; significant differences: *p < 0.05, **p < 0.01, ***p < 0.001, and ****p < 0.0001.

To accurately replicate the complex three‐compartmentalized architecture of the muscle‐tendon unit, a thorough investigation and optimization of the microfluidic wet‐spinning parameters were required. Ideally, the extrusion speed of the core–shell microfibers should not exceed the rotational speed of the collecting drum (**Figure** **2**A). Ensuring compliance with these parameters enabled the deposition of the m‐ink and t‐ink precisely on the same location of the collecting drum at each rotation. Conversely, if the RS of the drum was set lower than the microfiber extrusion rate, an inconsistent deposition pattern of the m‐ink and t‐ink occurred, subsequently yielding a scaffold that lacks the desired organization and compartmentalization. To identify a range of microfluidic spinning parameters for the fabrication of MTJ‐mimetic constructs, core–shell fibers were wet‐spun with different flow rates, and the diameter of the fibers were evaluated to quantify the speed of the extruded microfibers (Figure 2B and Figure S2A and Table S1 (Supporting Information). Comparative evaluation of such findings against the selected range of RS values resulted in the generation of a heatmap, delineating the available operational ranges for the fabrication of the targeted scaffold (Figure 2C). In Table S2 (Supporting Information), the tangential speed values corresponding to RS are reported. Also, In Figure S2B (Supporting Information), the corresponding heatmap plotting the fiber extruded speed versus the tangential speed values of the rotational drum are reported. Within the defined MTJ‐spinnability window, heterogeneous scaffolds could be fabricated, characterized by a controlled ratio of microfiber having different cores (Figure 2D). This could be achieved by using a constant translational speed in the x‐axis while simply tuning the extrusion time of the different inks. Furthermore, by modulating the extrusion time of the two different inks, it was possible to obtain scaffolds with distinct proportions of the m‐ink and t‐ink. Figure 2E presents a series of core–shell yarns showing a gradient descent decrease in t‐ink content accompanied by an incremental increase of m‐ink concentration. Such versatile technological features may be used to biofabricate samples with different cell ratios tailored to the specific tissue. Furthermore, the composition of the respective inks can be adjusted in alignment with the proliferation dynamics of muscle or tendon progenitor cells. Collectively, these characteristics validated the proposed system as a suitable platform for the biofabrication of highly aligned oriented tissues, such as skeletal muscles, tendons, and complex tissue interfaces as the MTJ.

### 2.3 Dependence of Core–Shell Microfiber Features on Drum RS

**Figure 3:**
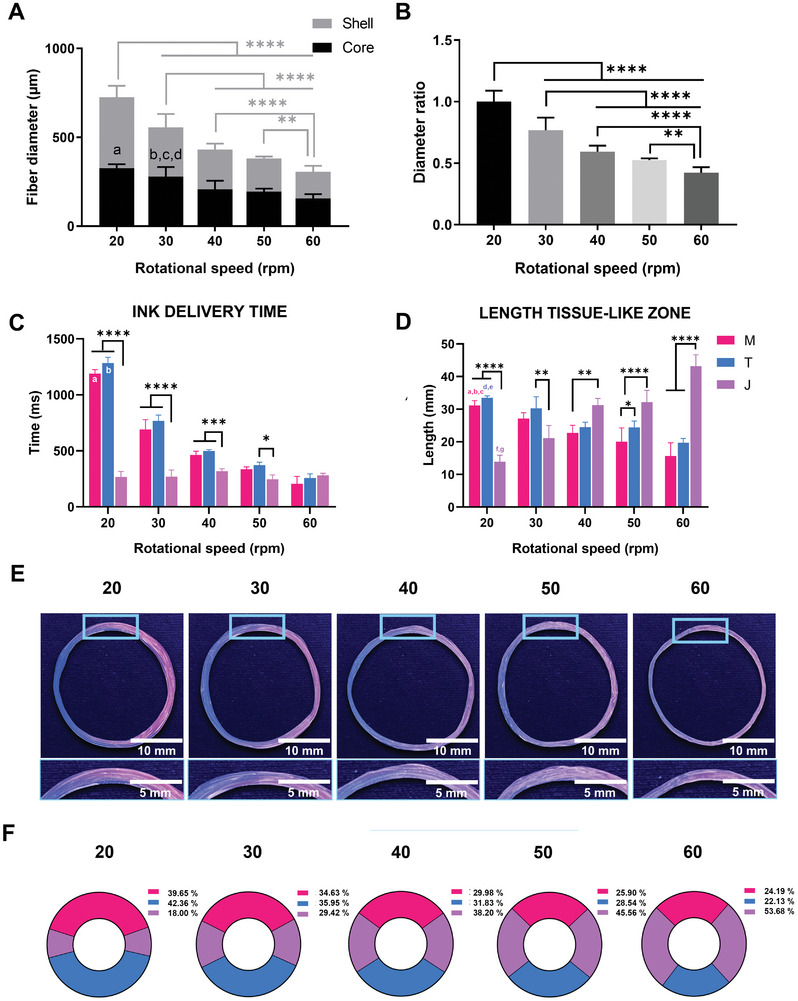
Evaluation of the effect of different rotational speeds (RS) (i.e., 20, 30, 40, 50, 60 rpm) on both the core–shell microfiber diameter and the myotendinous junction (MTJ)‐like scaffold compartmentalization. A) Core–shell microfibers diameter at selected RS. B) Diameter ratio of core–shell microfibers at selected RS. Fiber diameter is normalized to the diameter of core–shell microfibers wet‐spun at RS = 20 rpm, which is considered to be equivalent to the speed of the extruded microfibers (fiber speed, FS). C) Delivery time (DT) of muscle‐like ink(m‐ink), tendon‐like ink (t‐ink) and junction‐like (j‐ink) at the selected RS. D) Segment length related to m‐ink, t‐ink, and j‐ink at selected RS. E) Macroscopic images of MTJ‐like fiber wet‐spun at selected RS values. To identify the m‐ink and t‐ink, core‐inks were mixed with pink and blue‐fluorescent particles, respectively. F) Representative pie‐chart of the proportion of m‐ink, t‐ink, and j‐ink in MTJ‐scaffolds wet‐spun at selected RS. The core–shell fibers and the MTJ‐like core–shell yarns were obtained by using shell flow rate (*Q*_s_) = 320 µL min^−1^ and (*Q*_c_) = 160 µL min^−1^. *n* = 3; significant differences: *p < 0.05, **p < 0.01, ***p < 0.001, and ****p < 0.0001. A) *a* = **** 20 versus 40, 50, 60, *b* = ** 30 versus 40, *c* = *** 30 versus 50, *d* = **** 30 versus 60, D) *a* = **** 20 M versus 30 M versus 40 M versus 50 M versus 60 M, E) *a* = ** 20:M versus 40:M, *b* = *** 20:M versus 50:M, *c* = *** 20:M versus 60:M, *d* = ** 20:T versus 40:T, 20:T versus 50:T, *e* = **** 20:T versus 60:T, *f* = * 20:J versus 30:J, *g* = ****20:J versus 40:J, 20:J versus 50:J and 20:J versus 60:J.

The RS emerges as a pivotal factor when delineating the optimal parameter for the generation of MTJ‐like scaffolds. Specifically, spinning a core–shell yarn with different RS values influenced two primary biofabrication aspects: i) the core–shell fiber diameter and ii) the degree of the scaffold compartmentalization. Core–shell yarns were produced at different RS (i.e., 20, 30, 40, 50, 60 rpm), and subsequent evaluations were conducted on both the shell and core diameters. It was discerned that increasing the RS corresponded to a significant reduction in the diameters of both the shell (*D*_s_) and core (*D*_c_). In particular, we observed maximum values of *D*_s_ and *D*_c_ (*D*_s_ = 725.80 ± 60.47 µm, *D*_c_ = 326.90 ± 20.80 µm) for 20 rpm and a minimum value of *D*_s_ and *D*_c_ for 60 rpm (*D*_s_ = 306.60 ± 31.21 µm, *D*_c_ = 156.50 ± 22.37 µm) (**Figure** **3**A). Considering that a RS of 20 rpm corresponded to the extrusion rate of core–shell fibers for *Q*_s_ = 320 µL min^−1^ and *Q*_c_ = 160 µL min^−1^, higher RS values induced an additional mechanical stretching. This could be evidenced by the decrease in the overall microfiber diameter ratio (Figure 3B). Such phenomena, combined with the extrusion of core–shell microfibers through the coaxial nozzle, might also influence the reorganization of the core microstructure, thereby enhancing the alignment of the fibrinogen/alginate polymeric chains.^[^ ^24^ ^]^ The influence of the RS on the compartmentalization degree of MTJ‐like scaffolds was also investigated. Indeed, the selection of RS required to be matched with the m‐ink and t‐ink delivery time (DT). In this context, DT refers to the timespan time during the fabrication process when the m‐ink and the t‐ink are physically dispensed, from the initiation to the end of the delivery process.

In the optimal scenario—wherein the extruded core–shell microfibers adhered seamlessly to the circumference of the rotating drum—the combined DT of m‐ink and t‐ink should correspond to the duration required for the drum to undergo a full rotation. Figure 3C shows the DT of m‐ink (DT_m‐ink_), t‐ink (DT_t‐ink_), and DT of the junction ink (j‐ink, DT_j‐ink_) for each RS value. Here, DT_j‐ink_ refers to the transition time wherein either m‐ink or t‐ink is extruded, subsequently displacing t‐ink or m‐ink within the core‐delivery channel of the T‐junction. For each RS examined, the ink duration times revealed significant variations. Specifically, it was found that higher RS entailed a shorter DT_m‐ink_ and DT_t‐ink_, whereas DT_j‐ink_ remained constant (306.26 ± 33.09 ms). For RS set at 20 rpm, it was observed the longest ink DT (i.e., m‐ink = 1191.50 ± 44.33 ms with t‐ink = 1285.25 ± 30.72 ms). In contrast, the ink DT at 60 rpm was notably shorter (i.e., m‐ink = 206.00 ± 33.60 ms and t‐ink = 257.50 ± 44.33 ms). Additionally, for RS set at 20, 30, and 40 rpm, DT_m‐ink_ and DT_t‐ink_ are significantly longer than those of DTj_‐ink_. However, DTsj_‐ink_ at 50 and 60 rpm closely align with those of DT_m‐ink_ and DT_t‐ink_. Such differences in the DT, coupled with variations in the diameter of core–shell microfibers, reflected the segment lengths corresponding to muscle, tendon, and MTJ regions (Figure 3D). For the RS of 20 rpm, we measured the shortest segment length corresponding to j‐ink (13.00 ± 1.73 mm), compared to m‐ink at 33.50 ± 1.36 mm and t‐ink at 31.09 ± 0.53 mm. In contrast, at 60 rpm, j‐ink extends to its longest length at 43.17 ± 3.04 mm, while m‐ink and t‐ink measured 19.69 ± 3.53 mm and 15.63 ± 1.15 mm, respectively. The macroscopic evaluations of MTJ‐like scaffolds obtained by alternate wet‐spinning of m‐ink and t‐ink mixed with pink and blue‐fluorescent dyes, respectively, offered a visual confirmation of this phenomenon (Figure 3E). Higher RS generated scaffolds with a poor spatial compartmentalization of the inks. Quantitatively, scaffolds were characterized by an enlarged MTJ region relative to the entire construct and its t m‐ink‐ and t‐ink‐areas (Figure 3F). Conversely, scaffolds spun at lower RS exhibited a pronounced compartmentalization, where all three zones—, i.e., muscle, tendon, and MTJ—were distinctly demarcated. Consequently, proper tissue proportions were ensured without regions invading one another. This condition was considered desirable since each distinct section could then be properly analyzed independently and in relation to its adjacent tissue structures. Such high degree of compartmentalization was paramount for the development of an advanced platform that facilitates a comprehensive assessment of the muscle‐tendon unit.

### 2.4 C2C12 Myoblasts and NIH 3T3 Fibroblasts Alignment versus RS

**Figure 4:**
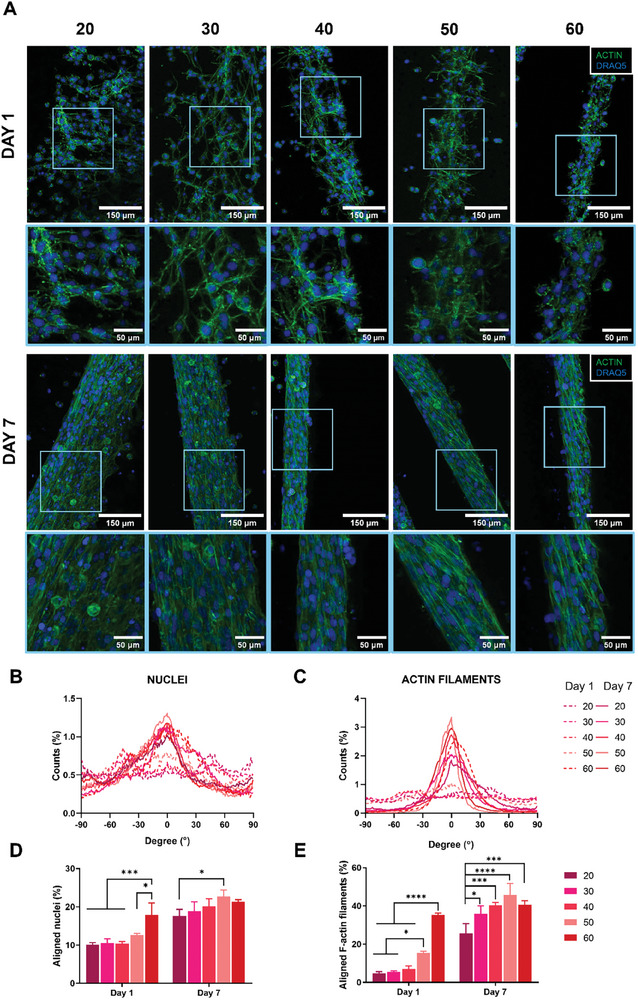
Assessment of C2C12 myoblast alignment in microfibers wet‐spun with different rotational speeds (RS) (i.e., 20, 30, 40, 50, 60 rpm) at day 1 and day 7 of cell culture. A) Representative confocal images of actin (green) and DRAQ5 (blue). Distribution plot of B) aligned nuclei and C) F‐actin filaments. 0° corresponds to the longitudinal axis of the microfiber. Percentage quantification of D) aligned nuclei and E) F‐actin filaments. The percentage was evaluated considering C2C12 myoblasts aligned within ± 10° of the longitudinal direction. *n* = 3; significant differences: *p < 0.05, **p < 0.01, ***p < 0.001, and ****p < 0.0001.

Uniaxial cellular orientation is paramount for the successful bioengineering of skeletal muscle, tendon, and, by extension, MTJ tissue structures. Such architectural organization is intrinsically linked to their physiological functions in vivo. For instance, skeletal muscle contraction relies on the parallel arrangement of myofibers, which is crucial for efficient and uniaxial force generation and locomotion.^[^ ^28^, ^29^, ^30^, ^31^ ^]^ Tendons transmit forces from muscles to bones. Such unidirectional force transmission necessitates a highly aligned 3D ECM network to efficiently transfer the mechanical load.^[^ ^32^, ^33^ ^]^ In light of these features, the orientation of tendons and muscle precursors cells is a key factor in the successful biofabrication of MTJ‐inspired scaffolds. To this aim, both C2C12 myoblasts and NIH3T3 fibroblasts were wet‐spun under varying RS, and the influence of RS on cellular alignment along to the microfiber axis was comprehensively assessed. At day 1, C2C12 myoblasts exhibited the sharpest peak of cell distribution plot at 60 rpm (**Figure** **4**A–E). At such RS value, it was also observed the highest percentage of aligned nuclei (17.93 ± 2.53%) and F‐actin filament (35.4 ± 0.71%). In contrast, cells displayed a more random orientation within the microfiber core at reduced speeds.

**Figure 5:**
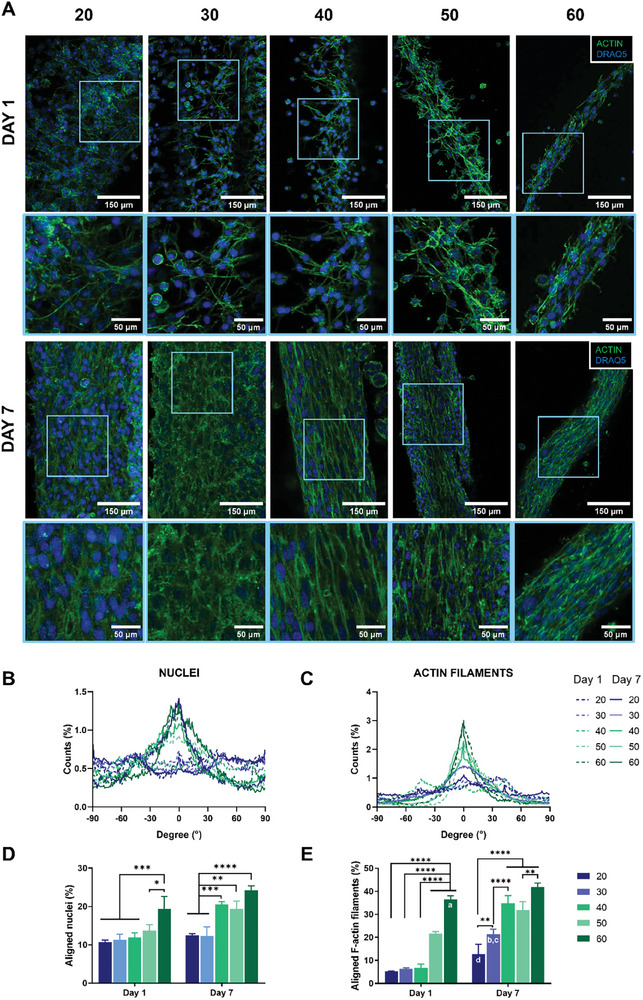
Assessment of NIH 3T3 fibroblasts alignment of microfibers wet‐spun with different rotational speeds (RS) (i.e., 20, 30, 40, 50, 60 rpm) at day 1 and day 7 of cell culture. A) Representative confocal images of actin (green) and DRAQ5 (blue). Distribution plot of B) aligned nuclei and C) F‐actin filaments. 0° corresponds to the longitudinal axis of the microfiber. Percentage quantification of D) aligned nuclei and E) F‐actin filaments. The percentage was evaluated considering NIH 3T3 fibroblasts aligned within ± 10° of the longitudinal direction. *n* = 3; significant differences: *p < 0.05, **p < 0.01, ***p < 0.001, and ****p < 0.0001. E) *a* = **** 50 versus 60, *b* = **** 30 versus 60, *c* = *** 30 versus 50, *d* = * 40 versus 60.

The observed phenomenon might be attributed to the dimensional characteristics of the microfibers, which imposed geometric constraints that progressively confined cells as the core diameter decreased, consequently influencing their elongation.^[^ ^34^, ^35^ ^]^ Furthermore, core–shell fibers spun at the same values of Q_s_ and Q_c_ and collected at higher RS might display an enhanced hydrogel matrix alignment, thus affecting cell anisotropic orientation.^[^ ^31^, ^36^ ^]^ Moreover, core–shell microfibers that underwent wet‐spinning at diminished rotational velocities remained immersed in the crosslinking bath for an extended duration. As a consequence, the alginate present in the core‐ink may undergo augmented crosslinking, culminating in a more rigid hydrogel matrix.^[^ ^37^ ^]^ Within such a network, cells might exhibit inhibited spreading and adopt a more rounded morphology.^[^ ^38^ ^]^ Furthermore, a denser crosslinking network results in smaller mesh and pore sizes in the hydrogel. This restricts the space available for cells to spread and extend processes like filopodia.^[^ ^39^ ^]^ Additionally, accessibility to binding sites might be limited, reducing the formation of focal adhesions, which are key structures in cell anchoring and spreading. Overall, such effect might have prevented cells from manifesting a discernible orientation along the microfiber's longitudinal axis. By day 7, a remarkable degree of cells alignment was detected across all RS, except for 20 rpm (17.66 ± 1.39% of nuclei and 24.51 ± 4.33% of f‐actin filaments oriented within ±10° along the microfiber longitudinal axis). Herein, it was observed that the core diameter had a negligible impact on cell alignment. This observation can be related to the inherent tendency of C2C12 myoblasts to initiate fusion, resulting in the formation of highly aligned myotubes.^[^ ^40^ ^]^ In addition, differentiated myoblasts might exert mechanical forces on the encapsulating hydrogel matrix, which can lead to localized matrix remodeling.^[^ ^41^ ^]^ Such effect could cause mechanical compaction of the hydrogel, thus reducing the overall core diameter and further enhancing cell orientation along the microfiber direction.^[^ ^42^ ^]^ At day 1, NIH 3T3 fibroblasts also exhibited pronounced alignment at elevated RS (i.e., 60 rpm), accounting for 19.39 ± 2.66% of nuclei 36.57 ± 1.20% F‐actin filament oriented closely to microfiber axis (**Figure** **5**A–E). At reduced speeds, a propensity for actin filopodia to disperse transversely was observed, and cell nuclei lacked a distinct orientation. At day 7, a sharper peak in both nuclei and F‐actin distribution and a higher cell alignment were observed for high RS (i.e., 40, 50, 60 rpm). Conversely, hydrogel yarns wet‐spun at lower RS (i.e., 20, 30 rpm, respectively) exhibited limited uniaxial‐oriented cell organization with lower values of nuclei (12.46 ± 0.33% for 20 rpm, 13.35 ± 1.89% for 30 rpm) and filament actin (12.72 ± 3.46% for 20 rpm, 21.33 ± 1.77% for 30 rpm) oriented within ±10° along the microfiber longitudinal axis. We speculated that a wider core diameter might not provide the proper geometric constraints for directing an anisotropic cellular alignment.^[^ ^43^ ^]^ Besides, the inherent rapid proliferative nature of the fibroblasts can cause a disordered architecture in the absence of stringent confinement. Additionally, the excessive production of ECM components, including collagen, could alter the natural architecture of the tissue, leading to the loss of the desired cellular alignment.^[^ ^44^ ^]^ The pronounced proliferative behavior might also cause the core diameter to partially expand, accommodating cellular growth but neglecting to maintain or control the intended directionality of cellular development and subsequent tissue formation. Notably, the trends in the cell alignments did not affect the cell survival rates, showing no significant differences in viability values for both C2C12 myoblasts and NIH 3T3 fibroblasts spun at different RS (Figure S3, Supporting Information). In conclusion, such comprehensive evaluation of the effect of different RS guided the determination of the optimal parameters for the MTJ‐scaffold biofabrication. Herein, an intermediate value, i.e., 40 rpm, emerged as optimal, striking a balance between well‐defined scaffold compartmentalization and ensuring significant alignment of both tendon and muscle precursor cells.

### 2.5 C2C12/NIH 3T3‐Laden Viability and Expression of Tissue‐Specific Markers

**Figure 6:**
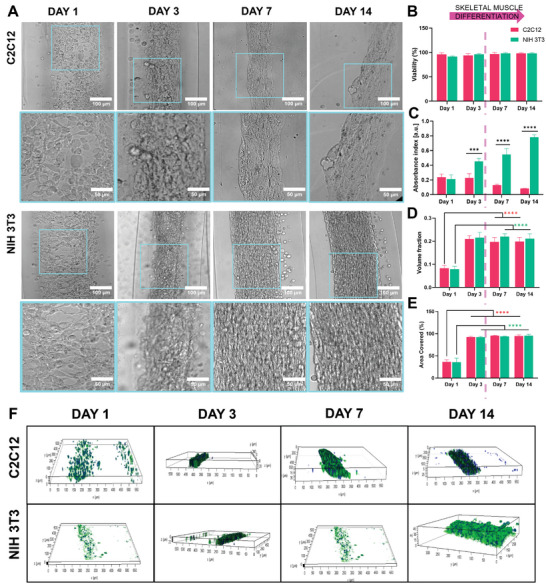
Assessment of proliferation of C2C12‐laden and NIH 3T3‐laden scaffolds at day 1, day 3, day 7, and day 14 of cell culture. A) Representative optical images. B) and viability, C) metabolic activity, D) volume fraction, E) percentage of area covered by cells. F) Representative 3D rendering of scaffold stained by actin (green) and DRAQ5 (blue). *n* = 4; significant differences: *p < 0.05, **p < 0.01, ***p < 0.001, and ****p < 0.0001.

**Figure 7:**
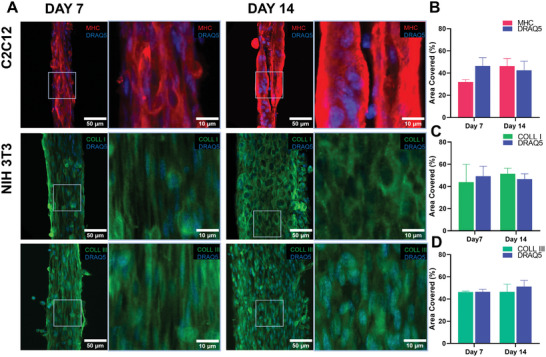
Assessment of functionality of C2C12‐laden and NIH 3T3‐laden scaffolds at day 7 and day 14 of cell culture. A) Representative confocal images of C2C12‐laden scaffolds stained with myosin heavy chain (MHC) antibody (red) and DRAQ5 (blue) and NIH 3T3‐laden scaffolds stained with collagen type I (COLL I) (green), collagen type III (COLL III) (green), and DRAQ5 (blue). Quantification of B) MHC, C) COLL I, D) COLL III together with cell nuclei (DRAQ5).

To validate the efficacy of the proposed biofabrication method for singularly engineering skeletal muscle and tendon tissues, C2C12 myoblasts and NIH 3T3 fibroblasts were wet‐spun separately, and cell‐laden scaffolds were cultured for 14 d. **Figure** **6**A shows the progress of cell elongation and growth within the core of the microfibers. Both types of constructs displayed high viability during the whole culture period, thus demonstrating that the engineered platform can equally maintain the in vitro survival rate of C2C12 myoblasts and NIH 3T3 fibroblasts, respectively, over 2 weeks of incubation (Figure 6B). Besides, metabolic assessment was also tested, revealing significant differences in the proliferative patterns inherent to each cell type (Figure 6C). Specifically, C2C12 myoblasts demonstrated a surge in proliferation until day 3, followed by a marked decline in metabolic activities. Indeed, this time point corresponded to the complete coverage of the inner core volume, as demonstrated by the observed plateau in both the volume fraction (Figure 6D) and the area (Figure 6E) occupied by cells. This stabilization in the cell growth indicated a saturation point in the colonization of the microfibers. Figure 6F further supports these findings by providing a 3D rendering that visually depicts the comprehensive coverage of the core by day 3 of culture. The achievement of the confluency corresponded to the cessation of myoblast proliferation, as the cells exited the cell cycle and began differentiating into mature myocytes, which can fuse into multinucleated myotubes.^[^ ^45^ ^]^ On the contrary, NIH 3T3 cells displayed a continuous increase in the metabolic activities despite switching into the muscle‐inducing differentiation medium. Fibroblasts are known to generally proliferate at high rates, a feature that reflects their role in key biological processes such as the repair of damaged tissue through the deposition of ECM components or the maintenance of tissue homeostasis.^[^ ^46^, ^47^, ^48^ ^]^ Evaluation of the proliferation trends of C2C12/NIH 3T3 encapsulated into the core–shell yarn was paramount to define the optimal wet‐spinning parameters to ensure an efficient co‐culture system. For instance, in skeletal muscle tissue engineering, the optimal scenario may involve fibroblasts offering structural support without hindering the myoblasts' growth and differentiation. Immunofluorescence analysis was performed to validate the potential of C2C12 myoblasts and NIH 3T3 fibroblasts to promote the maturation of skeletal muscle and tendon tissue, respectively (**Figure** **7**A). On day 14, myoblasts showed the formation of anisotropically oriented myotubes, exhibiting a full coverage of myosin heavy chain (MHC) expression (Figure 7B), a hallmark of skeletal muscle differentiation.^[^ ^49^ ^]^ Tenogenic ECM deposition was assessed through IF staining targeting relevant tenogenic proteins, specifically collagen type I and III. These types of collagens represent 60%–80% of dry tendon ECM components and play a crucial role in the healing and regeneration of the tissue.^[^ ^24^ ^]^ At day 7 and day 14, it was possible to observe abundant pericellular secretion of collagen type I and III, covering the majority of the inner core volume (Figure 7C,D). Given the preferential cell orientation and consequent collagen expression, we postulated that our platform could promote the aligned orientation and deposition of collagen type I and III, thus enabling the formation of an oriented matrix that closely mirrors the architecture of native tendon ECM.

### 2.6 Wet‐Spinning of Cell‐Laden MTJ‐Like Yarns

**Figure 8:**
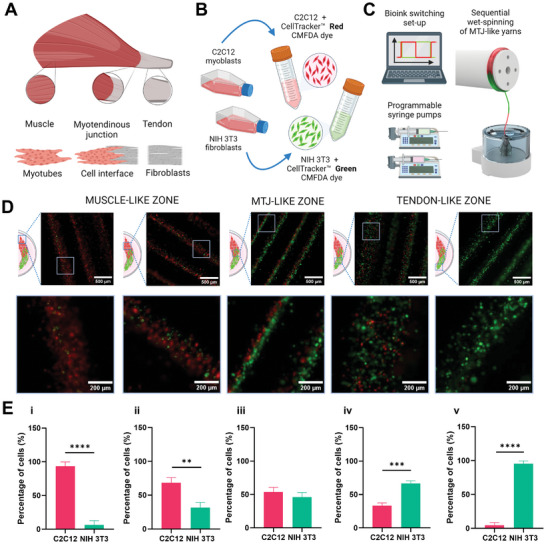
Schematics of the wet‐spinning process for the biofabrication of multicellular myotendinous junction (MTJ)‐like scaffolds. A) Native anatomy of the muscle‐tendon interface. B) Labeling process of C2C12 myoblasts and NIH 3T3 fibroblasts with viable red and green cell tracker, respectively. C) Sequential wet‐spinning process of cell‐laden hydrogel microfibers. D) Fluorescence images of C2C12 myoblasts and NIH 3T3 fibroblasts and their distribution within the microfiber core. E) Percentage of C2C12 myoblasts and NIH 3T3 fibroblasts in muscle‐ (i, ii), MTJ‐ (iii), and (iv, v) tendon‐like zones, respectively. *n* = 4; significant differences: *p < 0.05, **p < 0.01, ***p < 0.001, and ****p < 0.0001.

The successful modeling of the intricate biological architecture of the myotendinous junction stands as a significant milestone in the field of tissue engineering. The MTJ is a sophisticated nexus where muscle and tendon tissues converge (**Figure** **8**A).^[^ ^50^ ^]^ A multidisciplinary approach is crucial to successfully recapitulate its unique microstructural organization, as well as its cellular composition. In fact, the biofabrication of an engineered muscle‐tendon interface equivalent demands precise control over cellular placement and ECM deposition to ensure proper functional biomimicry. To fully mimic the biological composition of the MTJ, C2C12 myoblasts, and NIH 3T3 fibroblasts were wet‐spun in a sequential, continuous manner. The cellular distribution within the microfiber core was assessed by pre‐labeling C2C12 myoblasts and NIH 3T3 cells with red and green‐emitting viable cell trackers, respectively (Figure 8B,C).

Fluorescence imaging delineated a tri‐zonal cellular organization: a muscle‐like region predominantly occupied by C2C12 myoblasts, a MTJ region exhibiting a balanced distribution of NIH 3T3 fibroblasts and C2C12 myoblasts, and a tendon‐mimetic region predominantly comprising NIH 3T3 fibroblasts (Figure 8D,E). Such findings validated the selection of the biofabrication parameters, which effectively led to a multi‐cellular‐laden scaffold with a controlled cell distribution, compartmentalization, and patterns typical of the muscle‐tendon unit. Furthermore, the spatial fidelity of cells along the microfiber confirmed the effectiveness of the rapid Ca^2+^‐mediated crosslinking of alginate within the core–shell fibers, thus efficiently immobilizing the cell position during microfiber extrusion, which in turn prevented unwanted cell intermixing due to rapid switching of bioinks.

### 2.7 Immunocytochemistry Evaluation of Cell‐Laden MTJ‐Like Yarns

**Figure 9:**
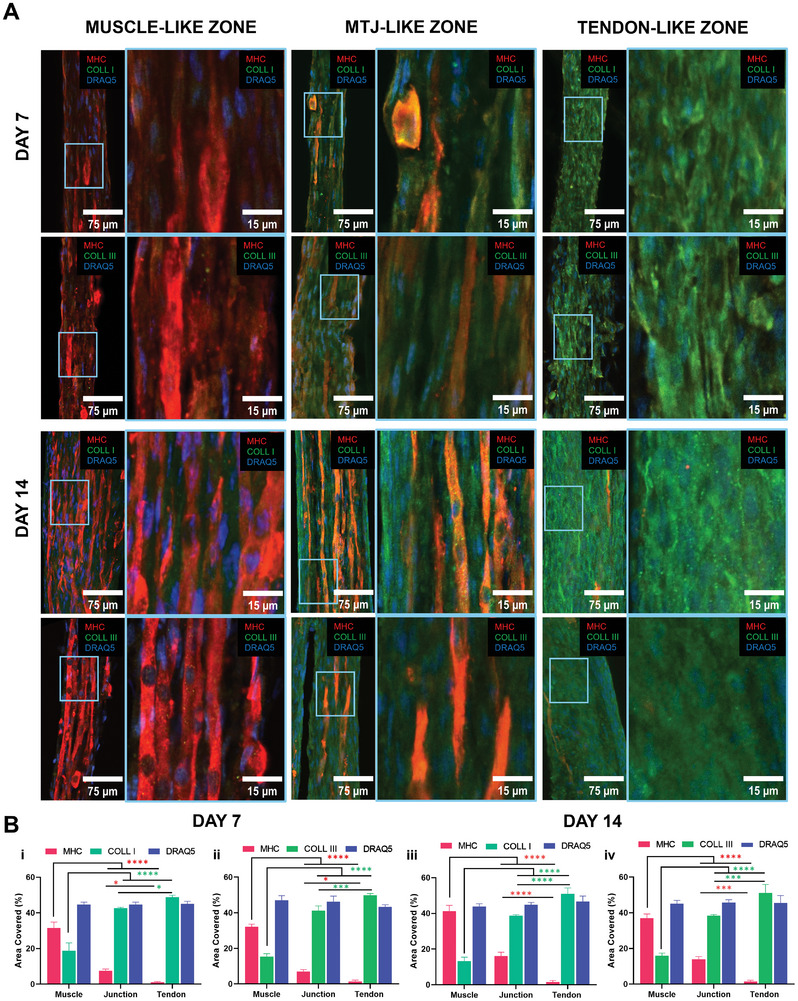
Representative confocal images of myotendinous junction (MTJ)‐like C2C12 myoblasts/NIH 3T3 fibroblasts yarns stained for myosin heavy chain (MHC) (red), collagen type I (COLLI) (green), collagen type III (COLL III) (green), and DRAQ5 (blue) at day 7 and day 14 of culture. Confocal images represent tissue‐specific regions: muscle, MTJ, and tendon. B) Quantification of MHC, COLLI (i, iii), COLLIII (ii, iv) and DRAQ5 in muscle, junction, and tendon regions of MTJ‐like yarns at (i, ii) 7 and (iii, iv) 14 d of culture. *n* = 4; significant differences: *p < 0.05, **p < 0.01, ***p < 0.001, and ****p < 0.0001.

In this study, MTJ‐like C2C12/NIH 3T3‐laden scaffolds were cultured under static conditions for 14 days. Immunolabeling evaluations were conducted to detect the localization of the selective expression of tissue‐specific markers (MHC, collagen type I, collagen type III, **Figure** **9**A). At day 7, three distinct regions emerged from immunofluorescence image analysis. The muscle‐mimetic region revealed the formation of myotubes characterized by the expression positive of MHC. No significant signal was detected in this region against collagen type I/III. At the level of the MTJ region, a transition zone was displayed, characterized by a collagen‐rich matrix encapsulating nascent myotubes. Lastly, in the tendon‐like region, a pronounced expression of type I and type III collagen was detected, while myotube presence was paltry. This phenomenon can be reasonably attributed to the dominant presence of NIH 3T3 fibroblasts in the specific area. We hypothesized that the prolific ECM production by the NIH 3T3 cells, coupled with their rapid proliferation, may inhibit the cell‐to‐cell contact of the sparse myoblasts, thus restricting myotube formation.^[^ ^51^ ^]^ On day 14, a more discernible zonal repartition could be identified due to the progression of tissue‐specific maturation. Specifically, the muscle‐mimetic region was characterized by an abundant array of parallel‐aligned, multinucleated, myosin‐positive myotubes. In the MTJ domain, a significant assembly of myotubes was intricately meshed within the connective tissue‐like matrix generated by the fibroblasts. Remarkably, the architecture of these myotubes mirrored the finger‐like protrusions of the skeletal muscle sarcolemma into the tenogenic tissue, a hallmark of the native MTJ.^[^ ^52^ ^]^ The replication of such interdigitating structure in vitro plays a crucial role in terms of muscle‐tendon unit functionality. Indeed, these projections increase the surface area of contact between the muscle and tendon, thus evenly distributing the mechanical load across the junction.^[^ ^53^ ^]^ Furthermore, they enhance the overall strength of the MTJ, minimizing the risk of detachment under high mechanical loads.^[^ ^54^ ^]^ Ultimately, the tendon‐mimetic region demonstrated a pronounced expression of collagen type I and III, indicative of a fully developed tenogenic extracellular matrix.^[^ ^55^ ^]^ Observations regarding expressions of tissue‐specific markers were further substantiated through the quantification of confocal images (Figure 9B). Notably, the highest percentage of area coverage by MHC was detected in the muscle region. Conversely, a high percentage of collagen area coverage was observed in the tenogenic region. The junction domain exhibited an intermediate value of area coverage between these two markers, characterized by a predominance of collagen embedding myotubes. Tensile‐mechanical evaluation of C2C12/NIH 3T3‐laden wet‐spun yarns was in line with the biological characterization of the heterogeneous scaffolds (Figure S4, Supporting Information). Indeed, MTJ‐like yarns demonstrated intermediate stiffness, combining properties of both cell types. These findings illustrated the positive impact of the coculture platform, enhancing mechanical properties through a collagen‐based matrix that supported myotube formation.

In this study, we introduced an innovative approach to cohesively integrate muscle and tendon tissues, mirroring the native continuity between these biological structures. Our methodology uniquely encapsulates both cell types within a single core in a gradient‐like pattern. This strategy can be considered an advancement from previous biofabrication methodologies that compartmentalized tendon and myoblast precursor cells. Indeed, the generation of such heterogeneous scaffolds mainly relied on using 3D printing technologies involving multi‐cartridge and multi‐nozzle systems for multi‐cellular and multi‐material extrusion.^[^ ^2^, ^56^ ^]^ Such techniques suffer from several manufacturing limitations, including time‐effectiveness, costs, complex 3D printing apparatus, and intricate scaffold design. Furthermore, they cannot fully recreate the MTU gradient biological composition. Additionally, the continuity of the scaffold and the integration at the juncture site between muscle and tendon often lack robustness, preventing the mutual and direct interaction between muscle and tendon precursor cells. With our approach, we fostered a graded environment wherein cells at transitional zones could directly communicate and migrate, thereby mimicking key features of the MTJ. Crucially, the tissue‐specific biological characteristics of both the tendon and muscle were successfully preserved. By optimizing the cell density ratio between NIH 3T3 fibroblasts and C2C12 myoblasts, coupled with proper formulation of the growth and differentiation medium, we ensured an efficient co‐culture model of these cells without negatively affecting their inherent behaviors. Indeed, we speculate that an unbalanced concentration of NIH 3T3 fibroblasts could potentially lead to a significant release of TGF‐β, potentially inhibiting myoblast differentiation and impacting both the quality and the extent of the myotube formation.^[^ ^57^, ^58^ ^]^ Besides, fibroblasts could engage in metabolic competition for glucose, potentially restricting the proliferation and survival of myoblasts.^[^ ^59^ ^]^ Contrarily, our multi‐cellular culture model unveiled the benefits of cellular interaction for specific tissue generation. Notably, the fibroblasts generated a well‐defined ECM, offering a collagen‐rich structural framework. Such biological scaffold delivered the necessary mechanical and biochemical signals, supporting myoblast differentiation and the subsequent formation of myotubes.^[^ ^60^ ^]^ Further, the potential paracrine emission of signaling moieties, such as the Fibroblast Growth Factor‐2, could have further augmented myoblast differentiation.^[^ ^61^ ^]^

## 3 Conclusions

In this work, we developed MTJ‐like hydrogel yarns via a novel microfluidics‐assisted 3D printing wet‐spinning technique combined with programmable syringe pumps to rapidly switch m‐ink and t‐ink. By fine‐tuning the wet‐spinning parameters, we obtained biomimetic, highly compartmentalized scaffolds that effectively promoted anisotropic alignment of both muscle and tendon precursor cells. The sequential wet‐spinning of C2C12 myoblasts and NIH 3T3 fibroblasts provided a controlled gradient pattern that effectively replicated the biological composition and arrangement of the muscle‐tendon interface. Localized expression of tissue‐specific markers identified muscle and tenogenic maturation in the designed scaffold regions. Furthermore, interdigitation of mature muscle myofibers within the tenogenic extracellular matrix was detected at the level of the transition zone. Such findings validate our biofabrication method as a potential engineering platform for the regeneration of the muscle‐tendon interface in vitro.

## 4 Experimental Section

### Materials and Cells

All chemicals were purchased from Merck (former Sigma‐Aldrich, Germany) and used without further purification unless otherwise stated. Low molecular and high molecular weight sodium alginates (LMW‐ALG, *M*w 33 kDa and HMW‐ALG, *M*w 1000 kDa, respectively) were kind gifts from FMC Biopolymers (USA). Calcium chloride (CaCl_2_) and sodium chloride (NaCl) were purchased from Eurochem BGD (Poland). (4‐(2‐hydroxyethyl)−1‐piperazine ethanesulfonic acid) (HEPES) was purchased from Roth GmbH (Germany), C2C12 myoblasts were a kind gift of Dr. Cesare Gargioli (Tor Vergata University of Rome, Italy), and NIH 3T3 fibroblasts were bought from ATTC (USA).

### 3D Bioprinting Microfluidics‐Assisted Wet‐Spinning Setup

Core–shell hydrogel microfibers were fabricated and 3D spatially deposited using a custom 3D wet‐spinning bioprinter. The whole platform consists of i) an extrusion system composed of a microfluidic printing head bearing a crosslinking bath microtank with a co‐axial nozzle (inner needle diameter = outer needle diameter = 500 µm) placed at the bottom of it for the immediate gelation of core/shell fibers, ii) a rotating drum collector (diameter = 25 mm, length = 180 mm), and iii) an X‐axis (travel range = 160 mm) for the sequential extrusion process. The entire system was controlled with an Arduino Mega board and a custom software developed in Python. Specifically, the microfluidic dispensing head was designed to provide a rapid switch among the m‐ink and t‐ink bioinks. The overall system was obtained by combining the MPH with two core‐delivery channels converging in a T‐junction (2 inlets, 1 outlet) and one shell‐delivery channel together with the co‐axial extruder system. The MPH was fabricated using micromilling technique engraving microfluidic channels into three‐layered 3‐mm‐thick polycarbonate sheets. T‐junction channels were milled with a 400×800 µm cross‐section. After machining, the three sheets were sonicated in isopropanol for 30 minutes and then sealed with a hot press at 130 °C for 50 min. The core–shell chip outlet was fluidically connected to the core‐extruder (metal nozzle with blunt tip, 21G) and shell‐extruder (produced with computerized numerical control (CNC) lathe‐based process). The core extruder and the shell extruder were mounted co‐axially. Finally, the microfluidic printing head was equipped with a crosslinking bath microtank and mounted on the bioprinter's X‐axis arm. The inlets of the printing head were connected to programmable microfluidic syringe pumps (neMESYS low pressure, Cetoni GmbH, Germany) by autoclavable Teflon tubings (ID = 800 µm).

### Fabrication of Acellular Core–Shell Fiber‐Based Yarns

Core–shell hydrogel fibers were extruded using the custom coaxial MPH. The core ink (core‐ink) was composed of 1.4% w/v fibrinogen from bovine plasma and 0.2% w/v LMW‐ALG in 25 × 10^−3^ m HEPES buffer solution containing 150 × 10^−3^ m NaCl. The shell ink (shell‐ink) was prepared by dissolving 2% w/v LMW‐ALG, 0.5% w/v HMW‐ALG, and 0.5% w/v alginate‐RGD (ALG‐RGD) in 25 × 10^−3^ m HEPES (alginate RGD synthesis is reported in Supporting Information). Both shell‐ink and core‐ink were supplied to the microfluidic printing head nozzle at selected flow rates (*Q*_s_ and *Q*_c_, respectively) to generate the co‐flow in the extrusion nozzle. The crosslinking bath microtank was then filled with 0.3 m CaCl_2_ solution prior to the bioinks extrusion. The gelation of the core–shell fibers occurred instantaneously in the proximity of the tip of the nozzle upon coming into contact with CaCl_2_. The resulting hydrogel fiber was initially pulled gently upwards with a tweezer until it reached the surface of the rotating drum to be continuously extruded while forming a bundle. Core–shell microfibers were wet‐spun at the selected RS. Each bundle consists of 20 core–shell fibers equivalent to 30 s of extrusion. Afterward, the samples were collected from the rotating drum. To crosslink the core‐ink, the bundles were incubated with 2 U mL^−1^ thrombin in 25 × 10^−3^ m HEPES buffer solution for 35 min at 37 °C.

### Assessment of the Core–Shell Microfiber Diameter and MTJ Spinnability

To characterize the core–shell fiber diameter, microfibers were wet‐spun at selected speeds, *Q*c and *Q*s. Images were taken with a confocal microscope (Leica TCS SP8, Germany) and analyzed with ImageJ software (National Institute of Health (NIH), USA). To facilitate the identification of the core and shell compartments, the 0.2% w/v of LMW‐ALG in the core‐ink was replaced with alginate conjugated with fluorescein isothiocyanate (ALG‐FITC) (Alginate‐FITC synthesis is reported in Supporting Information). During the microscopical data acquisition, samples were kept in 0.05 m CaCl_2_ solution dissolved in 25 × 10^−3^ m HEPES.

The fiber extrusion speed (Table S1 and Figure S2a, Supporting Information) was calculated according to the volumetric equivalence (Equation 1):

(1) $$\mathrm{Extrusion}\;\mathrm{speed}\;\mathrm{of}\;\mathrm{core}-\mathrm{shell}\;\mathrm{fibers}=\frac{Q}{A}$$

where *Q* is the sum of the *Q*_s_+*Q*_c,_ and *A* is the area of the microfiber cross‐section. To investigate the spinnability of muscle‐tendon junction (MTJ)‐like scaffolds, the ratio of the fiber extrusion speed to the tangential speed of the rotating drum was calculated. These ratios served as critical indicators of the spinnability of the scaffolds and were assigned values on a scale from 1 to 0, with the lowest ratios corresponding to 1 (indicative of optimal spinnability) and the highest ratios assigned a value of 0 (representing reduced spinnability). Intermediate values were scaled proportionately. These ratios were subsequently visualized on a heatmap to effectively depict the spinnability windows for the fabrication of MTJ‐like scaffolds.

### Cell Culture and Expansion

C2C12 myoblasts and NIH 3T3 fibroblasts were cultured in high glucose DMEM (HG DMEM) GlutaMAX supplement (Gibco_,_ Thermo Fisher Scientific, USA) supplemented with 10% heat‐inactivated fetal bovine serum (FBS, Gibco, Thermo Fisher Scientific, USA), 100 IU mL^−1^ penicillin and 100 mg mL^−1^ streptomycin (P/S) (Gibco, Thermo Fisher Scientific, USA) at 37 °C in 5% CO_2_ and 95% air atmosphere. Cells were detached and expanded at 50% cell confluency using trypsin‐EDTA solution (Gibco, Thermo Fisher Scientific, USA).

### Biofabrication of C2C12/NIH 3T3‐Cell‐Laden Yarns

C1C12‐laden and NIH 3T3‐laden, along with MTJ‐like C2C12/NIH 3T3‐laden yarns, were fabricated following the procedure outlined in Section 4.3 properly adapted for cell culture conditions. The core‐ink, shell‐ink, and the crosslinking CaCl_2_ solution were syringe‐filtered. The core‐ink was mixed with 10 × 10^6^ C2C12 myoblasts mL^−1^ and 10 × 10^6^ NIH 3T3 fibroblasts mL^−1^ or to obtain the m‐ink and t‐ink, respectively. Prior to use, the microfluidic printing head and tubing were first washed with 70% ethanol solution and ultimately rinsed with sterile dH2O. In all cellular experiments, flow rates were set as *Q*_c_ = 160 µL min^−1^ and *Q*_s_ = 320 µL min^−1^, respectively. For the fabrication of MTJ‐like C2C12/NIH 3T3‐laden yarns, the RS of the drum was set to 40 rpm, while DT for t‐ink and m‐ink was fixed at 750 ms. After core‐ink crosslinking in thrombin solution (2 U mL^−1^, HEPES 25 × 10^−3^ m), C1C12‐laden, NIH 3T3‐laden, and MTJ‐like C2C12/NIH 3T3‐laden yarns were cultured as stated in Section 4.5. After 4 d of culture, the growth medium was switched to skeletal muscle differentiation‐inducing medium by replacing the FBS supplement with 2% horse serum.

### Cell Alignment Assessment

C2C12 myoblasts and NIH 3T3 fibroblasts alignment was investigated by staining cell F‐actin filaments and nuclei. Cell‐laden scaffolds were fixed with 4 % paraformaldehyde for 30 minutes and then washed thrice with 25 × 10^−3^ m HEPES. Then, samples were permeabilized with a solution of 0.3% (v/v) Triton X‐100 dissolved in 25 × 10^−3^ m HEPES for 15 min. Constructs were then incubated in 1% (w/v) bovine serum albumin in 25 × 10^−3^ m HEPES for 30 min to inhibit the non‐specific binding. As a post‐blocking step, Alexa Fluor 488 phalloidin (1:40, Invitrogen) in 25 × 10^−3^ m HEPES was added for 40 min at room temperature. Finally, cell nuclei were stained with Draq5 solution (1:1000, Thermo Fisher Scientific, USA) in 25 × 10^−3^ m HEPES and incubated for 15 min, protected from light. After final washing, samples were visualized using a confocal microscope (Leica TCS SP8, Germany). Cell alignment was analyzed from confocal images by measuring the orientation of the nuclei and F‐actin filaments (ImageJ software). The area covered by cells was also quantified using ImageJ by designating regions of interest (ROI) within different fibers (*n* = 3).

### Cells Viability Assessment

The cell viability of biofabricated yarns was evaluated using LIVE/DEAD assay (Thermo Fisher Scientific, USA). Cell‐laden yarns were washed in 25 × 10^−3^ m HEPES and treated with LIVE/DEAD working solution for 20 min at room temperature. Herein, calcein was used to stain alive cells in green, while ethidium homodimer was employed to mark dead cells in red. Subsequently, scaffolds were washed in 25 × 10^−3^ m HEPES and imaged with a fluorescent microscope (Leica TCS SP8, Germany) at wavelengths corresponding to fluorophores of interest. Number of alive and dead cells was calculated from fluorescence images using the counting algorithm of ImageJ on green and red channels of three different areas of three independent samples. The viability was calculated according to Equation 2:

(2) $$\%\;\mathrm{viability}\;=\;100\;\%\frac{\left(\mathrm{Number}\;\mathrm{of}\;\mathrm{cells}\right)}{\left(\mathrm{Number}\;\mathrm{of}\;\mathrm{live}\;\mathrm{cells}+\;\mathrm{number}\;\mathrm{of}\;\mathrm{dead}\;\mathrm{cells}\right)}$$

### Cells Proliferation Assessment

Cell‐laden yarns were washed with HG DMEM GlutaMAX w/o FBS and transferred to new wells containing 1 mL of pre‐incubated medium. Subsequently, 200 µL of MTS (Cell Titer 96 Aqueous One Solution Cell Proliferation Assay, Promega, USA) was added, and the samples were incubated at 37 °C in 5% CO_2_ and 95% air atmosphere for 45 minutes. The reaction was stopped by adding 250 µL of 10% sodium dodecyl sulfate (SDS) and shaking for 30 min. Quadruplicate aliquots (100 µL each) of the supernatant were transferred to a 96‐well plate, and the optical density was registered at *λ* = 490 nm.

### Evaluation of the Cell Volume Fraction

Cell‐laden scaffolds were fixed with 4 % paraformaldehyde for 30 min and then washed thrice with 25 × 10^−3^ m HEPES. Then, samples were permeabilized with a solution of 0.3% (v/v) Triton X‐100 dissolved in 25 × 10^−3^ m HEPES for 15 min. Finally, cell nuclei were stained with Draq5 solution (1:1000, Thermo Fisher Scientific, USA) in 25 × 10^−3^ m HEPES and incubated for 15 min, protected from light. After final washing, samples were visualized using a confocal microscope (Leica TCS SP8, Germany). Confocal images were processed using ImageJ software. Volume fraction was calculated according to Equation 3:

(3) $$\mathrm{Volume}\;\mathrm{fraction}=\frac{n^{\circ }\mathrm{nuclei}\;\left(\mathrm{ROI}\right)\;x\;\mathrm{volume}\;\mathrm{of}\;\mathrm{nuclei}}{\mathrm{ROI}\;\mathrm{volume}}$$

where ROI is the region of interest.

### Cells Fluorescence Labeling

To evaluate the cellular distribution within the MTJ‐like scaffolds, C2C12 myoblasts and NIH 3T3 fibroblasts were fluorescently labeled using CellTracker CM‐DiI Red and CellTracker Green (Invitrogen, Thermo Fisher Scientific, USA), respectively. In particular, C2C12 myoblasts were trypsinized and harvested by centrifugation. Then, the supernatant was aspirated, and the cell pellet was gently resuspended into CellTracker CM‐DiI and kept at 4 °C for 5 min. Then, cells were transferred in the incubator at 37 °C under 5% CO_2_ and 95% air atmosphere for 15 min. Subsequently, the cell‐tracker solution was removed by centrifugation. NIH 3T3 fibroblasts were resuspended in the CellTracker Green Working Solution after detachment and centrifugation. Cells were incubated for 30 min at 37 °C in 5% CO_2_ and 95% air atmosphere. Labeled cells were gently mixed with syringe‐filtered core‐ink and wet‐spun in a sequential manner. MTJ‐like cell‐laden scaffolds were visualized with a fluorescent microscope (Leica TCS SP8, Germany) at wavelengths corresponding to the fluorescence dyes of interest.

### MHC, Collagen Type I, and Collagen Type III Immunocytochemistry

MHC staining was used to detect the degree of differentiation of myotubes, while Collagen type I (COLLI) and III (COLLIII) expression was assessed to evaluate the ECM deposition. Cell‐laden scaffolds were fixed with 4 % paraformaldehyde for 30 min, followed by triple washing using 25 × 10^−3^ m HEPES. To ensure the antibody penetration, the alginate shell was eliminated via a 3 h incubation at 37 °C in an ethylenediaminetetraacetic acid (EDTA)‐saturated solution containing 0.04% (w/v) alginase. Permeabilization was achieved by treating the samples with 0.3% (v/v) Triton X‐100 in 25 × 10^−3^ m HEPES (containing 0.04% alginase) for 2 h at 37 °C. Post Triton X‐100 removal with 25 × 10^−3^ m HEPES, blocking was performed using 1% (w/v) bovine serum albumin in 25 × 10^−3^ m HEPES with 0.04% alginase for 2 h at 37 °C. Samples were then subjected to incubation with mouse‐derived Anti‐Myosin (Skeletal) antibody (MF 20, DSHB) (1:1) and rabbit‐derived anti‐collagen I (1:100) and anti‐collagen III (1:100) overnight at 4 °C. After washing, the constructs were incubated with Alexa Fluor 555 anti‐mouse secondary antibody produced in goat solution (1:500, Invitrogen) for 3 h at room temperature. Subsequently, cell nuclei were stained with DAPI solution (1:1000) and incubated for 15 minutes in the darkness at room temperature. After washing, the cell‐laden constructs were imaged under a confocal microscope (Nikon, A1R). Confocal images were processed using ImageJ software.

### Statistical Analysis

All measurements were made in triplicates on at least three different samples. Data are reported as mean values ± standard deviation. Two‐way ANOVA with Tukey's multiple comparison tests was performed using GraphPad Prism analysis software (GraphPad Software, USA) to investigate statistical differences between data populations. Differences are displayed as statistically significant when *p*‐values (*p*) ≤ 0.05. Statistically significant values are presented as *p ≤ 0.05, **p ≤ 0.01, ***p ≤ 0.001, and ****p ≤ 0.0001.

## Conflict of Interest

The authors declare no conflict of interest.

## Supporting information



Supporting Information

## Data Availability

The data that support the findings of this study are available from the corresponding author upon reasonable request.
