# Supplementary material for: Automated Microfluidics‐Assisted Hydrogel‐Based Wet‐Spinning for the Biofabrication of Biomimetic Engineered Myotendinous Junction
Source: Adv Healthc Mater. 2024 Sep 23;13(32):2402075. doi: 10.1002/adhm.202402075 (PMC11670271; doi:10.1002/adhm.202402075)
Supplement: Supplementary file 1 — Supporting Information [file ADHM-13-0-s001.docx]

**Supporting Information**

**Automated Microfluidics-Assisted Hydrogel-Based Wet-Spinning for the Biofabrication of Biomimetic Engineered Myotendinous Junction**

Marina Volpi^1^, Alessia Paradiso^1^, Ewa Walejewska^1^, Cesare Gargioli^2^, Marco Costantini^3^*, and Wojciech Swieszkowski^1^*

^1^ Faculty of Materials Science and Engineering, Warsaw University of Technology, Warsaw 02-507, Poland

^2^ Department of Biology, University of Rome Tor Vergata, Rome 00133, Italy

^3^ Institute of Physical Chemistry, Polish Academy of Sciences, Warsaw 01-224, Poland

***Corresponding Authors: Dr. Marco Costantini** e-mail: [mcostantini@ichf.edu.pl](mailto:mcostantini@ichf.edu.pl),
**Prof. Wojciech Swieszkowski** e-mail: [wojciech.swieszkowski@pw.edu.pl](https://d.docs.live.net/8b18c5468f9cca2a/Desktop/wojciech.swieszkowski@pw.edu.pl)

**Keywords:** microfluidics-assisted wet-spinning, 3D bioprinting, biofabrication, hydrogel fibers, myotendinous junction, skeletal muscle tissue engineering

**1 Results
1.1 Rheological characterization**

As shown in **Figure S1**, the viscosity of the core-ink was unmeasurable within the shear rate range of 0.1 to 25 (1/s), due to the inability to reach steady flow equilibrium. The shell-ink demonstrated shear viscosity values ~200-fold higher than the core-ink. Furthermore, both the shell-ink and core-ink exhibited constant shear viscosity behavior at increasing shear rate values, thus indicating their Newtonian fluid features.


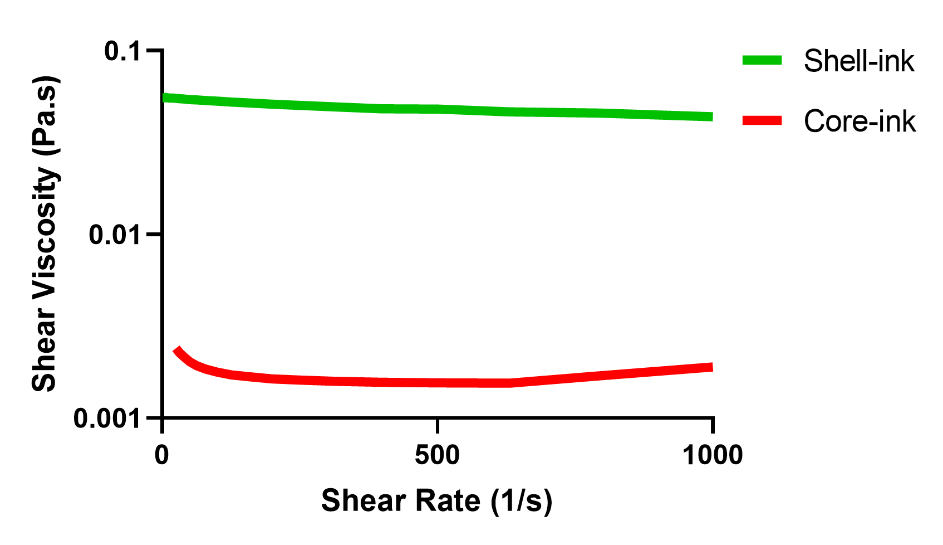


**Figure S1**. Shear viscosity of the shell-ink (alginate 3% w/v)) and core-ink (fibrinogen 1.4% w/v+alginate 0.2% w/v) at different shear rate values.

**1.2 Parametrical conversion

Table 1** presents the flow rates for shell and core, respectively, along with their associated extrusion speeds, while **Table 2** lists the values for rotational speed and corresponding tangential speed.

**Table S1**. Shell and core flow rate values and the corresponding extrusion speed values

| **Shell flow rate**  **(µL/min)** | 80 | 480 | 320 | 160 | 80 |
| --- | --- | --- | --- | --- | --- |
| **Core flow rate (µL/min)** | 40 | 240 | 160 | 80 | 40 |
| **Extrusion speed (mm/s)** | 13.30 ± 2.19 | 19.55 ± 1.91 | 25.16 ± 2.19 | 32.80 ± 1.71 | 40.33 ± 4.50 |

**Table S2.** Rotational speed values and the corresponding tangential speed values

| **Rotational speed (rpm)** | 10 | 20 | 30 | 40 | 50 | 60 |
| --- | --- | --- | --- | --- | --- | --- |
| **Tangential speed (mm/s)** | 13.08 | 26.17 | 39.26 | 52.35 | 65.44 | 78.53 |


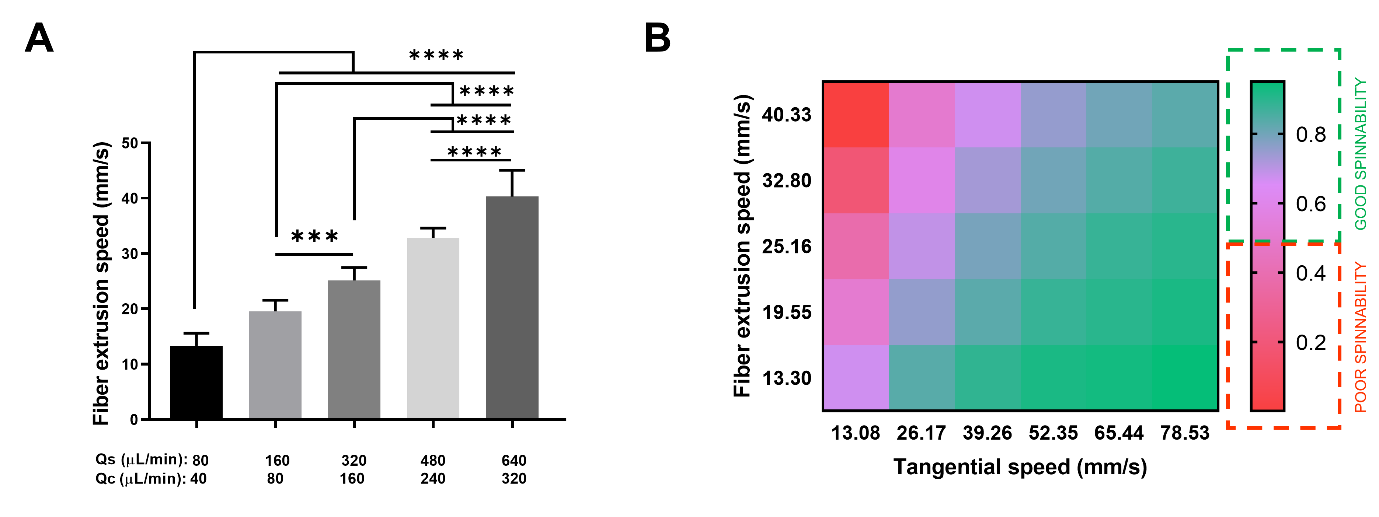


**Figure S2.** Investigation of myotendinous junction (MTJ)-like spinnability. A) Fiber extrusion speed values for different shell and core flow rate. B) Heatmap of MTJ-spinnability showing the combination of tangential speed and fiber extrusion speed for the fabrication of MTJ-like scaffolds. n = 4; significant differences: *p < 0.05, **p < 0.01, ***p < 0.001, and ****p < 0.0001.

**1.3 Viability of C2C12 and NIH 3T3 fibroblasts versus RS**

No significant differences in viability values was detected for both C2C12 myoblasts and NIH3T3 fibroblasts wet-spun under different RS (**Figure S3**). Such findings indicated that the RS, the diameter of core-shell fibers, and the alignment trends did not affect cell survival rates at day 1 and day 7 of cell culture.

**
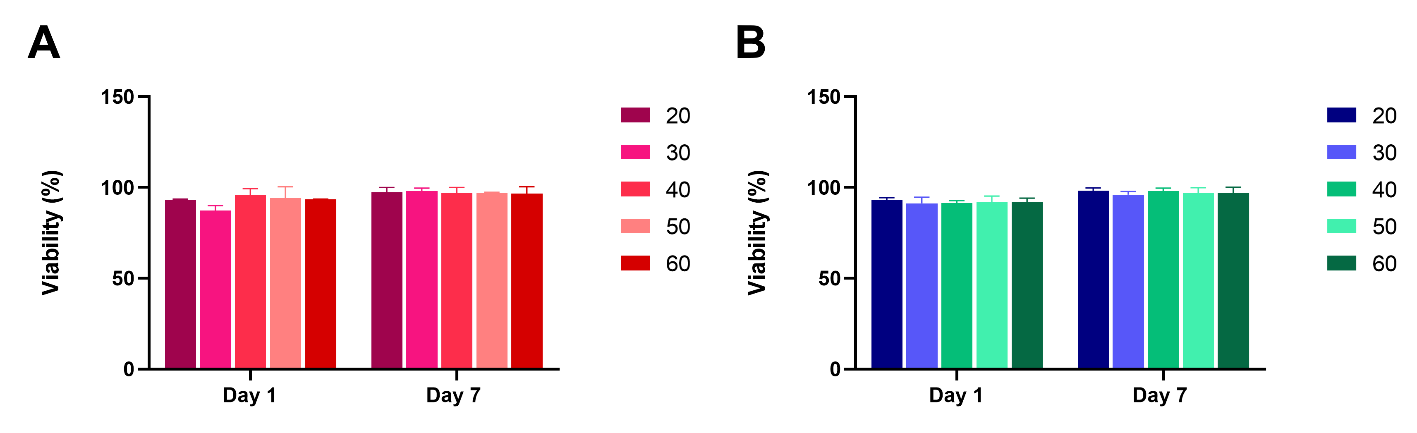
**

**Figure S3.** Viability of A) C2C12 myoblasts and B) NIH 3T3 fibroblasts wet-spun with different rotational speed (RS) (i.e., 20, 30, 40, 50, 60 rpm) at day 1 and day 7 of cell culture.

**1.4 Mechanical characterization**

Mechanical properties of control (no cell-laden), C2C12-laden, NIH 3T3-laden, C2C12/NIH 3T3-laden wet-spun yarns were assessed at different cell culture time points and are presented in **Figure S4**. Results showed a decrease in the mechanical properties over the culture time due to the alginate and fibrinogen degradation. Statistical analysis revealed no significant differences in mechanical properties between the cell-laden and control samples, thus indicating that cell encapsulation did not negatively affect the stiffness of the core-shell hydrogel yarns. At day 14, C2C12-laden yarns exhibited the lowest mechanical stiffness (40.33 ± 4.10 kPa). We speculated that this outcome could be caused to the contractile forces exerted by C2C12 myoblasts on the fibrinogen-based matrix. Conversely, NIH 3T3-laden fibroblasts displayed higher Young’s modulus values (54.60 ± 5.83 kPa), potentially due to collagen production within the core-shell fibers. Specifically, C2C12/NIH 3T3-laden yarns demonstrated intermediate mechanical behavior (50.33 ± 6.54 kPa), showing a blend of properties from myoblast and fibroblast-only samples. Such findings confirmed the positive effect of the co-culture platform, showing an increase in mechanical properties due to the collagen-based matrix, which provided an essential support to the myotubes formation.


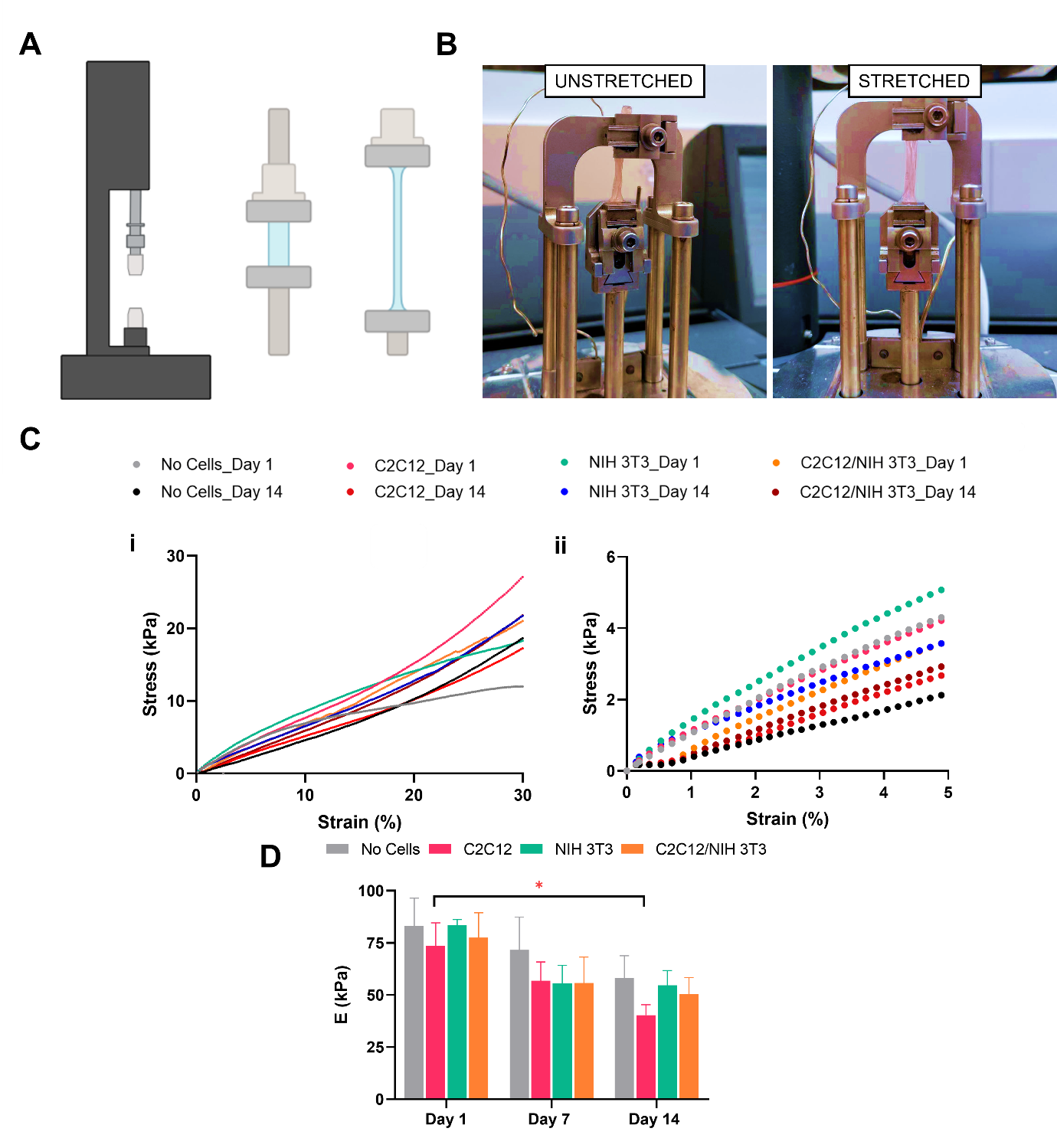


**Figure S4.** Assessment of mechanical properties of control (no cell-laden), C2C12-laden, NIH 3T3-laden, C2C12/NIH 3T3-laden wet-spun yarns at different cell culture time points (i.e., day 1, day 7, day 14). A) Schematics of tensile mechanical test performed on control/cell-laden wet-spun samples. B) Macroscopic images of a cell-laden wet-spun sample under unstretched and stretched conditions. C) Representative Stress-Strain curves at i) 30% and ii) 5% of elongation (datas related to day 7 are not shown). D) Young’s modulus (E) values. n = 4; significant differences: *p < 0.05, **p < 0.01, ***p < 0.001, and ****p < 0.0001.

**2. Materials and methods**

**2.1 Synthesis of alginate-FITC derivative**

Alginate-FITC derivative was synthesized through a two-step reaction. Briefly, 1 wt % sodium alginate solution was mixed with 1-Ethyl-3-(3-dimethylaminopropyl)carbodiimide (EDC, 50mg) and N-hydroxysuccinimide (NHS, 30 mg) for the activation of carbonyl groups on alginate in pH 4.9 sodium acetic buffer for 30 min, followed by addition of 1,6-diaminohexane (60 mg) for another 4 hours. The mixture was precipitated in 2-propanol to remove the unreacted diamine. The alginate-amine derivative reacted with fluorescein isothiocyanate (1 mg) in sodium carbonate buffer (pH 9.0) for 4 hours and was precipitated in acetone. Finally, the product was dialyzed against deionized water for three days and then freeze-dried.

**2.2 Alginate RGD synthesis**

To prevent the collapse of myofibers by ensuring mild cell attachment at the core-shell interface, Alginate-RGD was replaced with HM ALG at the same concentration (0.5% w/v) in the shell composition. Alginate-RGD was synthesized following previously published protocols. Briefly, 1-Ethyl-3-(3-dimethylaminopropyl) carbodiimide and N-Hydroxy sulfo succinimide were reacted with a 1% (w/v) alginate solution in a 2-ethanesulfonic acid (MES) buffer (0.1 M MES, 0.3 M NaCl, pH 6.5). Lyophilized GRGDSP peptides (ProteoGenix, France) were added to the solution at a concentration of 20 mg/g alginate and allowed to react overnight at room temperature. The peptide-modified alginate was subsequently purified by dialysis (3500 MWCO) at 4°C and lyophilized for further use.

**2.3 Rheological characterization**The rheological properties were assessed using a Kinexus Pro rotational rheometer (Malvern Panalytical Ltd) equipped with a double-gap geometry system, featuring diameters of 24 mm and 27 mm, at a controlled temperature (i.e., 25 °C). Viscosity measurements for both the shell-ink and core-ink were conducted in a shear rate-controlled mode, spanning shear rates from 0,1-1000 1/s.

**2.4 Mechanical characterization**Mechanical properties of control (no cell-laden), C2C12-laden, NIH 3T3-laden, C2C12/NIH 3T3-laden wet-spun yarns were assessed using Q800 (TA Instruments, USA) Dynamic Mechanical Analysis (DMA) instrument equipped with tension clamps. All the samples were stretched at a constant deformation rate of 5 %/min until yield with a preload set to 0.001 N. Young’s moduli were calculated as the slope in the 0–5% strain linear region from the obtained stress-strain curves.
